# Crystal structure of 2-cyano-1-methyl­pyridinium bromide

**DOI:** 10.1107/S2056989015019167

**Published:** 2015-10-17

**Authors:** Vu D. Nguyen, Cameron A. McCormick, Robert A. Pascal, Joel T. Mague, Lynn V. Koplitz

**Affiliations:** a Department of Chemistry, Loyola University, New Orleans, LA 70118, USA; b Department of Chemistry, Tulane University, New Orleans, LA 70118, USA

**Keywords:** crystal structure, salt, 2-cyano-1-methyl­pyridinium bromide

## Abstract

In the title mol­ecular salt, C_7_H_7_N_2_^+^·Br^−^, all the non-H atoms lie on crystallographic mirror planes. The packing consists of (010) cation–anion layers, with the cations forming dimeric units *via* very weak pairwise C—H⋯N inter­actions. Weak C—H⋯Br inter­actions link the cations to the anions.

## Related literature   

For structures of other salts of the 2-cyano-1-methyl­pyridinium cation, see: Koplitz *et al.* (2012 ▸); Kammer *et al.* (2013 ▸); Vaccaro *et al.* (2015 ▸). For structures of salts of the isomeric 2-cyano­anilinium cation, see: Oueslati *et al.* (2005 ▸); Cui & Wen (2008 ▸); Zhang, L. (2009 ▸); Zhang, Y. (2009 ▸); Cui & Chen (2010 ▸); Vumbaco *et al.* (2013 ▸).
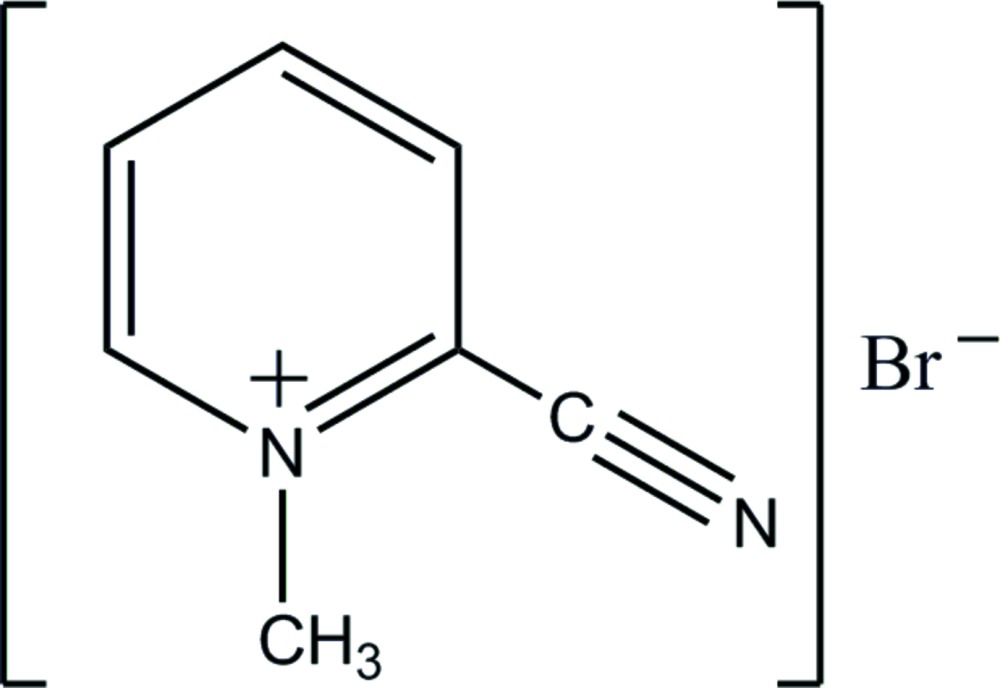


## Experimental   

### Crystal data  

C_7_H_7_N_2_^+^·Br^−^
*M* *_r_* = 199.06
Monoclinic, 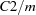

*a* = 13.3039 (12) Å
*b* = 6.5892 (6) Å
*c* = 9.3753 (8) Å
β = 92.419 (1)°
*V* = 821.13 (13) Å^3^
*Z* = 4
Mo *K*α radiation
μ = 4.93 mm^−1^
*T* = 150 K
0.20 × 0.15 × 0.06 mm

### Data collection  

Bruker SMART APEX CCD diffractometer
Absorption correction: multi-scan (*TWINABS*; Sheldrick, 2009 ▸) *T*_min_ = 0.44, *T*_max_ = 0.74
22367 measured reflections
1179 independent reflections
1084 reflections with *I* > 2σ(*I*)
*R*_int_ = 0.021

### Refinement  

*R*[*F* ^2^ > 2σ(*F* ^2^)] = 0.023
*wR*(*F* ^2^) = 0.048
*S* = 1.02
1179 reflections
62 parameters
H-atom parameters constrained
Δρ_max_ = 0.51 e Å^−3^
Δρ_min_ = −0.44 e Å^−3^

### 

Data collection: *APEX2* (Bruker, 2014 ▸); cell refinement: *SAINT* (Bruker, 2014 ▸); data reduction: *SAINT* and *CELL_NOW* (Sheldrick, 2008*a* ▸); program(s) used to solve structure: *SHELXT* (Sheldrick, 2015*a* ▸); program(s) used to refine structure: *SHELXL2014* (Sheldrick, 2015*b* ▸); molecular graphics: *DIAMOND* (Brandenburg & Putz, 2012 ▸); software used to prepare material for publication: *SHELXTL* (Sheldrick, 2008*b* ▸).

## Supplementary Material

Crystal structure: contains datablock(s) global, I. DOI: 10.1107/S2056989015019167/hb7523sup1.cif

Structure factors: contains datablock(s) I. DOI: 10.1107/S2056989015019167/hb7523Isup2.hkl

Supporting information file. DOI: 10.1107/S2056989015019167/hb7523Isup3.cml

**Fig. 1.**
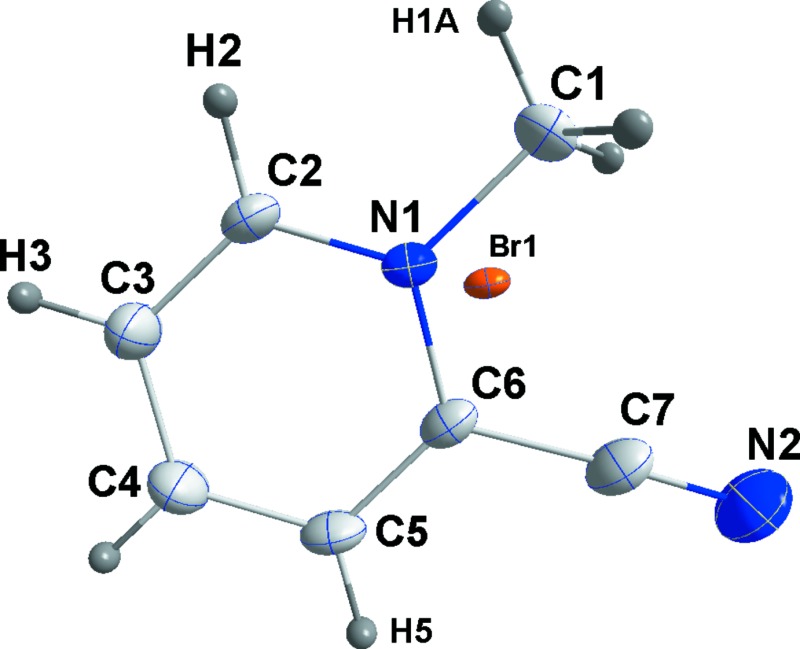
The title compound with labeling scheme and 50% probability ellipsoids.

**Fig. 2.**
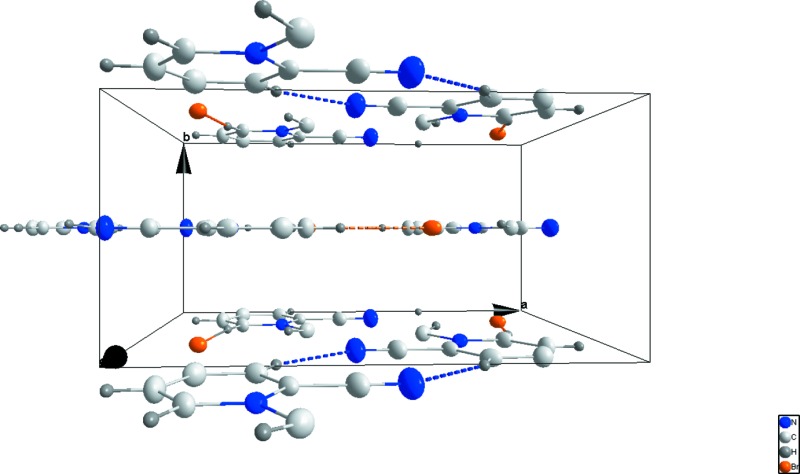
Packing viewed down the c axis showing the layer structure.

**Fig. 3.**
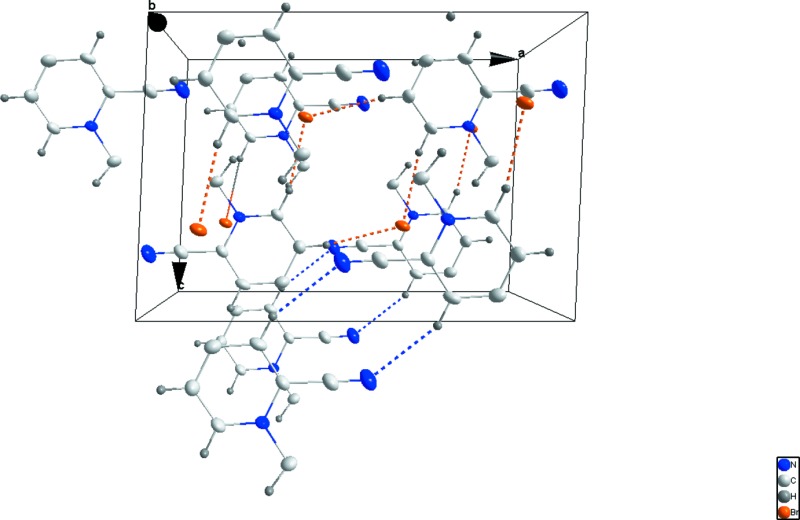
Packing viewed down the b axis showing the weak C—H···N (blue dotted lines) and C—H···Br (orange dotted lines) interactions.

. DOI: 10.1107/S2056989015019167/hb7523fig1.tif
The title compound with labeling scheme and 50% probability ellipsoids.

c . DOI: 10.1107/S2056989015019167/hb7523fig2.tif
Packing viewed down the *c* axis showing the layer structure.

b . DOI: 10.1107/S2056989015019167/hb7523fig3.tif
Packing viewed down the *b* axis showing the weak C—H⋯N (blue dotted lines) and C—H⋯Br (orange dotted lines) inter­actions.

CCDC reference: 1430625

Additional supporting information:  crystallographic information; 3D view; checkCIF report

## Figures and Tables

**Table 1:** Hydrogen-bond geometry (, )

*D*H*A*	*D*H	H*A*	*D* *A*	*D*H*A*
C5H5N2^i^	0.95	2.66	3.549(3)	155
C1H1*A*Br1^ii^	0.98	2.96	3.876(2)	156
C2H2Br1^ii^	0.95	2.66	3.586(2)	166
C3H3Br1^iii^	0.95	2.77	3.711(2)	170

Symmetry codes: (i) 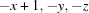; (ii) 
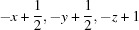
; (iii) 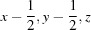.

## supplementary crystallographic information

### S1. Comment

The cation in the title compound has crystallographically imposed mirror symmetry with the methyl H atoms slightly disordered about the mirror. The packing thus consists of cation/anion layers (Fig. 2) with the cations forming dimeric units *via* weak, pairwise C5—H5···N2 interactions (Fig. 3 and Table 1). Within the layers weak C—H···Br interactions tie the cations and anions together (Fig. 3 and Table 1).

### S2. Experimental

2-Cyanopyridine (4.04 g, 38.8 mmol) was first melted in a warm water bath and then dissolved in toluene (15 ml). Gaseous bromomethane was condensed (roughly 5 ml, 170 mmol) and added to this solution slowly. The reaction mixture was thoroughly mixed to yield a light amber homogenous solution and left to evaporate slowly. Light yellow shiny flakes of 2-cyano-1-methylpyridinium bromide (m.p. 196.4–197.4 C) were collected by vacuum filtration.

### S3. Refinement

H-atoms attached to carbon were placed in calculated positions (C—H = 0.95 - 0.98 Å). All were included as riding contributions with isotropic displacement parameters 1.2 - 1.5 times those of the attached atoms. Trial refinements with the single-component reflection file extracted from the full dataset with *TWINABS* and with the full, 2-component reflection file indicated the former refinement to be superior.

### Crystal data

**Table:**

C_7_H_7_N_2_^+^·Br^−^	*F*(000) = 392
*M**_r_* = 199.06	*D*_x_ = 1.610 Mg m^−^^3^
Monoclinic, *C*2/*m*	Mo *K*α radiation, λ = 0.71073 Å
*a* = 13.3039 (12) Å	Cell parameters from 9936 reflections
*b* = 6.5892 (6) Å	θ = 2.2–29.1°
*c* = 9.3753 (8) Å	µ = 4.93 mm^−^^1^
β = 92.419 (1)°	*T* = 150 K
*V* = 821.13 (13) Å^3^	Block, colourless
*Z* = 4	0.20 × 0.15 × 0.06 mm

### Data collection

**Table:**

Bruker SMART APEX CCD diffractometer	1179 independent reflections
Radiation source: fine-focus sealed tube	1084 reflections with *I* > 2σ(*I*)
Graphite monochromator	*R*_int_ = 0.021
Detector resolution: 8.3660 pixels mm^-1^	θ_max_ = 29.1°, θ_min_ = 2.2°
φ and ω scans	*h* = −18→18
Absorption correction: multi-scan (*TWINABS*; Sheldrick, 2009)	*k* = −8→8
*T*_min_ = 0.44, *T*_max_ = 0.74	*l* = −12→12
22367 measured reflections	

### Refinement

**Table:**

Refinement on *F*^2^	Primary atom site location: structure-invariant direct methods
Least-squares matrix: full	Secondary atom site location: difference Fourier map
*R*[*F*^2^ > 2σ(*F*^2^)] = 0.023	Hydrogen site location: inferred from neighbouring sites
*wR*(*F*^2^) = 0.048	H-atom parameters constrained
*S* = 1.02	*w* = 1/[σ^2^(*F*_o_^2^) + (0.0234*P*)^2^] where *P* = (*F*_o_^2^ + 2*F*_c_^2^)/3
1179 reflections	(Δ/σ)_max_ = 0.001
62 parameters	Δρ_max_ = 0.51 e Å^−^^3^
0 restraints	Δρ_min_ = −0.44 e Å^−^^3^

### Special details

**Table:**

Experimental. The diffraction data were obtained from 3 sets of 400 frames, each of width 0.5° in ω, colllected at φ = 0.00, 90.00 and 180.00° and 2 sets of 800 frames, each of width 0.45° in φ, collected at ω = -30.00 and 210.00°. The scan time was 20 sec/frame. Analysis of 1897 reflections having I/σ(I) > 13 and chosen from the full data set with *CELL_NOW* (Sheldrick, 2008a) showed the crystal to belong to the monoclinic system and to be twinned by a 180 ° rotation about *a**. The raw data were processed using the multi-component version of *SAINT* under control of the two-component orientation file generated by *CELL_NOW*.
Geometry. All e.s.d.'s (except the e.s.d. in the dihedral angle between two l.s. planes) are estimated using the full covariance matrix. The cell e.s.d.'s are taken into account individually in the estimation of e.s.d.'s in distances, angles and torsion angles; correlations between e.s.d.'s in cell parameters are only used when they are defined by crystal symmetry. An approximate (isotropic) treatment of cell e.s.d.'s is used for estimating e.s.d.'s involving l.s. planes.
Refinement. Refinement of *F*^2^ against ALL reflections. The weighted *R*-factor *wR* and goodness of fit *S* are based on *F*^2^, conventional *R*-factors *R* are based on *F*, with *F* set to zero for negative *F*^2^. The threshold expression of *F*^2^ > σ(*F*^2^) is used only for calculating *R*-factors(gt) *etc*. and is not relevant to the choice of reflections for refinement. *R*-factors based on *F*^2^ are statistically about twice as large as those based on *F*, and *R*- factors based on ALL data will be even larger. H-atoms attached to carbon were placed in calculated positions (C—H = 0.95 - 0.98 Å). All were included as riding contributions with isotropic displacement parameters 1.2 - 1.5 times those of the attached atoms. Trial refinements with the single-component reflection file extracted from the full dataset with *TWINABS* and with the full, 2-component reflection file indicated the former refinement to be superior.

### Fractional atomic coordinates and isotropic or equivalent isotropic displacement parameters (Å^2^)

**Table:**

	*x*	*y*	*z*	*U*_iso_*/*U*_eq_	Occ. (<1)
N1	0.29873 (14)	0.0000	0.32868 (18)	0.0177 (4)	
N2	0.53530 (17)	0.0000	0.1900 (3)	0.0399 (6)	
C1	0.36322 (18)	0.0000	0.4616 (2)	0.0239 (5)	
H1A	0.3212	0.0177	0.5440	0.036*	0.5
H1B	0.4118	0.1116	0.4584	0.036*	0.5
H1C	0.3993	−0.1293	0.4701	0.036*	0.5
C2	0.19853 (17)	0.0000	0.3369 (2)	0.0218 (5)	
H2	0.1698	0.0000	0.4280	0.026*	
C3	0.13619 (18)	0.0000	0.2157 (2)	0.0269 (5)	
H3	0.0652	0.0000	0.2229	0.032*	
C4	0.17863 (19)	0.0000	0.0830 (3)	0.0273 (5)	
H4	0.1368	0.0000	−0.0017	0.033*	
C5	0.28191 (18)	0.0000	0.0749 (2)	0.0237 (5)	
H5	0.3120	0.0000	−0.0152	0.028*	
C6	0.34090 (17)	0.0000	0.1994 (2)	0.0198 (4)	
C7	0.44985 (19)	0.0000	0.1969 (3)	0.0280 (5)	
Br1	0.36532 (2)	0.5000	0.29528 (2)	0.02252 (9)	

### Atomic displacement parameters (Å^2^)

**Table:**

	*U*^11^	*U*^22^	*U*^33^	*U*^12^	*U*^13^	*U*^23^
N1	0.0207 (9)	0.0176 (8)	0.0152 (9)	0.000	0.0033 (7)	0.000
N2	0.0292 (12)	0.0484 (15)	0.0431 (14)	0.000	0.0139 (10)	0.000
C1	0.0261 (12)	0.0271 (12)	0.0182 (11)	0.000	−0.0025 (9)	0.000
C2	0.0220 (11)	0.0245 (11)	0.0196 (11)	0.000	0.0076 (8)	0.000
C3	0.0219 (11)	0.0337 (13)	0.0251 (12)	0.000	0.0018 (9)	0.000
C4	0.0300 (13)	0.0314 (13)	0.0203 (11)	0.000	−0.0022 (9)	0.000
C5	0.0315 (13)	0.0232 (11)	0.0170 (10)	0.000	0.0085 (9)	0.000
C6	0.0207 (11)	0.0178 (10)	0.0216 (11)	0.000	0.0080 (8)	0.000
C7	0.0274 (13)	0.0294 (13)	0.0280 (13)	0.000	0.0104 (10)	0.000
Br1	0.02982 (14)	0.02142 (12)	0.01687 (12)	0.000	0.00747 (8)	0.000

### Geometric parameters (Å, º)

**Table:**

N1—C2	1.339 (3)	C2—H2	0.9500
N1—C6	1.357 (3)	C3—C4	1.388 (3)
N1—C1	1.482 (3)	C3—H3	0.9500
N2—C7	1.141 (3)	C4—C5	1.379 (3)
C1—H1A	0.9800	C4—H4	0.9500
C1—H1B	0.9800	C5—C6	1.379 (3)
C1—H1C	0.9800	C5—H5	0.9500
C2—C3	1.378 (3)	C6—C7	1.451 (3)
			
C2—N1—C6	120.12 (19)	C2—C3—H3	120.5
C2—N1—C1	119.61 (18)	C4—C3—H3	120.5
C6—N1—C1	120.28 (18)	C5—C4—C3	119.5 (2)
N1—C1—H1A	109.5	C5—C4—H4	120.2
N1—C1—H1B	109.5	C3—C4—H4	120.2
H1A—C1—H1B	109.5	C6—C5—C4	119.1 (2)
N1—C1—H1C	109.5	C6—C5—H5	120.4
H1A—C1—H1C	109.5	C4—C5—H5	120.4
H1B—C1—H1C	109.5	N1—C6—C5	120.9 (2)
N1—C2—C3	121.25 (19)	N1—C6—C7	117.8 (2)
N1—C2—H2	119.4	C5—C6—C7	121.3 (2)
C3—C2—H2	119.4	N2—C7—C6	177.7 (3)
C2—C3—C4	119.1 (2)		
			
C6—N1—C2—C3	0.000 (1)	C1—N1—C6—C5	180.000 (1)
C1—N1—C2—C3	180.000 (1)	C2—N1—C6—C7	180.000 (1)
N1—C2—C3—C4	0.000 (1)	C1—N1—C6—C7	0.000 (1)
C2—C3—C4—C5	0.000 (1)	C4—C5—C6—N1	0.000 (1)
C3—C4—C5—C6	0.000 (1)	C4—C5—C6—C7	180.0
C2—N1—C6—C5	0.000 (1)		

### Hydrogen-bond geometry (Å, º)

**Table:**

*D*—H···*A*	*D*—H	H···*A*	*D*···*A*	*D*—H···*A*
C5—H5···N2^i^	0.95	2.66	3.549 (3)	155
C1—H1*A*···Br1^ii^	0.98	2.96	3.876 (2)	156
C2—H2···Br1^ii^	0.95	2.66	3.586 (2)	166
C3—H3···Br1^iii^	0.95	2.77	3.711 (2)	170

Symmetry codes: (i) −*x*+1, −*y*, −*z*; (ii) −*x*+1/2, −*y*+1/2, −*z*+1; (iii) *x*−1/2, *y*−1/2, *z*.
